# Splicing Programs and Cancer

**DOI:** 10.1155/2012/269570

**Published:** 2011-10-24

**Authors:** Sophie Germann, Lise Gratadou, Martin Dutertre, Didier Auboeuf

**Affiliations:** ^1^Université de Lyon, 28 rue Laënnec, 69008 Lyon, France; ^2^Inserm U1052, 28 rue Laënnec, 69008 Lyon, France; ^3^CNRS UMR5286, 28 rue Laënnec, 69008 Lyon, France; ^4^Centre de Recherche en Cancérologie de Lyon, Centre Léon Bérard, 28 Rue Laënnec, 69008 Lyon, France

## Abstract

Numerous studies report splicing alterations in a multitude of cancers by using gene-by-gene analysis. However, understanding of the role of alternative splicing in cancer is now reaching a new level, thanks to the use of novel technologies allowing the analysis of splicing at a large-scale level. Genome-wide analyses of alternative splicing indicate that splicing alterations can affect the products of gene networks involved in key cellular programs. In addition, many splicing variants identified as being misregulated in cancer are expressed in normal tissues. These observations suggest that splicing programs contribute to specific cellular programs that are altered during cancer initiation and progression. Supporting this model, recent studies have identified splicing factors controlling cancer-associated splicing programs. The characterization of splicing programs and their regulation by splicing factors will allow a better understanding of the genetic mechanisms involved in cancer initiation and progression and the development of new therapeutic targets.

## 1. Introduction

**Figure 1:**
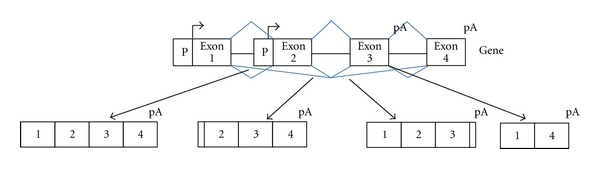
Genes are an assemblage of exons that can be differentially selected through the use of alternative promoters (P), alternative polyadenylation sites (pA), and alternatively spliced exons.

Each cellular program results from the expression of gene networks or transcriptional programs that are under the control of transcription factors. However, human genes can no longer be considered as simple functional units producing a single transcript. Rather, human genes are an assemblage of exons that can be differentially selected through the use of alternative promoters, alternative polyadenylation sites, and alternatively spliced exons (Figure 1). Genome-wide analyses of splicing based on ESTs (expressed sequence tags), splicing sensitive microarrays, or deep sequencing data sets have revealed that most, if not all human genes can generate different transcripts with different exon content and there are at least 10 times more mRNAs than genes [1–4]. It is now widely accepted that different cell types not only differ because they express different sets of genes but also because genes produce different splicing variants depending on cell type [3, 5–10]. Furthermore, coordinated regulation of alternative splicing of gene products within gene networks plays a key role during differentiation [11–13]. Therefore, an emerging model is that each cell type at a specific developmental stage is characterized by splicing programs that together, with other layers of gene expression programs (e.g., transcriptional programs), determine the precise nature of their transcriptome and therefore their proteome.

**Figure 2:**
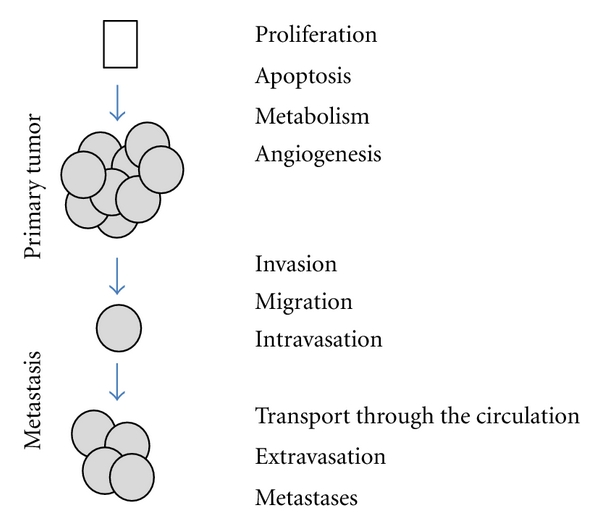
Tumor cells are able to adapt and to evolve. Tumor cells that proliferate develop mechanisms to escape apoptosis and control of their environment. Some tumor cells stimulate angiogenesis or degrade the extracellular matrix, migrate and colonize other tissues to form metastasis.

Tumor cells are able to adapt and evolve. Indeed, tumor cells that proliferate develop mechanisms to escape control by their environment. Some tumor cells stimulate angiogenesis or degrade the extracellular matrix, migrate and colonize other tissues to form metastasis (Figure 2) [14]. Gene expression regulation is obviously playing a critical role in this phenotypic plasticity. It is now widely accepted that many transcription factors are altered during tumor initiation and progression. Alterations can occur at the gene level (mutations, misexpression, etc.) or because signaling pathways controlling the activity of transcription factors are altered [14]. Collectively, these alterations result in the changes in transcriptional programs and therefore cellular programs. For example, it has been shown that misregulation of transcription factors, like TWIST that are involved in embryonic development, can induce the epithelial-mesenchymal transition (EMT) that is implicated in the conversion of early-stage tumors into invasive malignancies [15].

**Figure 3:**
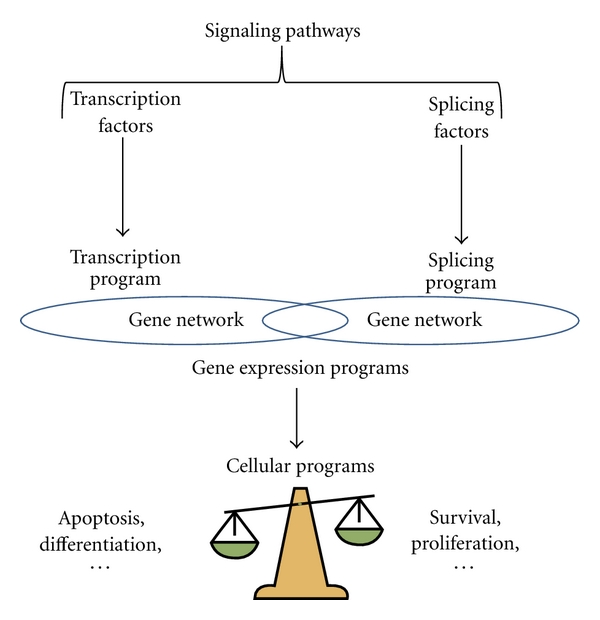
Cellular programs depend on gene expression programs that result from both transcriptional and splicing programs. Transcriptional and splicing programs are under the control of transcription and splicing factors, respectively. Mutations or gene expression alteration of transcription and/or splicing factors can contribute to tumor initiation and progression. The activity of transcription and splicing factors is also under the control of signaling pathways that can be altered in tumor initiation and progression.

Because alternative splicing permits the generation of protein isoforms having different biological activities, it is likely that alterations of splicing regulation participate in the phenotypic plasticity of tumor cells. In this context, many splicing variants have been found to be misregulated in cancers [16–24]. However, one major challenge now is to better characterize the splicing programs contributing to specific cancer-associated phenotypes and to identify the splicing factors that control such splicing programs. Indeed, the recognition of exons and introns relies on degenerated sequences at the boundaries between them (splicing sites) that are recognized by the spliceosome, as well as on splicing regulatory sequences located within exons and introns that are recognized by accessory factors or splicing factors (e.g., SR and hnRNP proteins) [16, 17, 19, 20, 25–27]. Depending on their nature and the position of their binding sites, splicing factors can either strengthen or inhibit the splice sites recognition by the spliceosome and can therefore enhance or repress the inclusion of alternative exons. Like transcription factors control transcriptional programs by controlling the expression of gene networks, splicing factors control splicing programs by controlling alternative splicing of gene networks (Figure 3). While several excellent reviews on splicing and cancer have been recently published [16–24, 28–32], our aim in this paper was to discuss recent genome-wide analyses of alternative splicing in cancer indicating that splicing programs controlled by splicing factors play a major role in cellular programs and tumor progression.

## 2. Large-Scale Analyses of Alternative Splicing in Cancer

There are now numerous studies reporting on splicing alterations in many cancers (see above). However, understanding of the role of alternative splicing in cancer is now reaching a novel level, thanks to the use of new tools including splicing-sensitive microarrays, allowing the analysis of splicing variant expression at a large-scale level. As summarized in Table 1, tumors from breast, lung, digestive tract, and brain have been extensively analyzed thanks to these tools. Although the number of splicing alterations depends on the study design and cancer type, and even though extensive validations using different approaches have generally not been done, it appears that splicing alterations affect gene networks participating in key cellular programs.

For example, we recently used the exon arrays from Affymetrix to search for potential splicing variants associated with different metastatic properties in the clinically relevant 4T1 mouse model of spontaneous breast cancer metastasis [35]. The 4T1 mouse model comprises four syngenic tumor lines (67NR, 168FARN, 4T07, and 4T1) that have differential metastatic behavior: the 67NR cell line forms primary carcinomas when implanted into the mouse mammary fat pad, and no tumor cells are detected at distant tissue; the 168FARN cell line forms primary carcinomas with extensions to local lymph nodes; the 4T07 and 4T1 cell lines generate micrometastases and macroscopic metastases, respectively, in the lungs. By comparing the transcriptome at the exon level in primary tumors generated from each cell line, we identified 679 splicing variants that were differentially expressed in primary tumors with different metastatic abilities. Many of the splicing variations identified in the 4T1 model affected genes involved in cellular morphology and movement, which suggests that splicing may play a role in these cellular activities that are highly relevant to tumor progression. In addition, many splicing events identified in the primary tumors were conserved during evolution and were found in normal tissues, demonstrating that at least a large subset of splicing variations during tumor progression are not due to aberrant splicing. Importantly, some of the splicing variants identified in mouse tumors giving rise to metastases were linked with poor prognosis (shorter metastasis-free survival) in a large cohort of breast cancer patients. Using the primary tumors generated from the 168FARN, 4TO7, and 4T1 cell lines, Bemmo and collaborators also identified genes that are differentially spliced in association with the metastatic ability of tumors [36]. The misregulated genes were involved in cell growth and proliferation, cell death, cellular development, and cellular movement.

Lapuk and collaborators also identified 156 genes being differentially spliced when comparing 26 breast cancer cell lines representing the luminal, basal, and claudin-low subtypes of primary breast tumors, and five nonmalignant breast cell lines [34]. Functional annotation analyses of the regulated genes showed preferential enrichment of biological processes related to cytoskeleton and actin. In addition, this study revealed that many of these splicing events are regulated by the FOX2 (RBM9) splicing factor. This is of particular interest as Venables and collaborators also identified a large number of differentially spliced genes when comparing human breast and ovarian tumors to normal tissues that are under the control the FOX2 splicing factor that the authors showed to be downregulated in ovarian cancer and to be altered at the splicing level in breast cancer samples [33]. Interestingly, several studies suggest that FOX2 is a critical regulator of a splicing network. Indeed, by integrating binding specificity with phylogenetic conservation and splicing microarray data from 47 tissues and cell lines, Zhang and collaborators found thousands of FOX tissue-specific targets [50]. Yeo and collaborators also constructed an RNA map of FOX2-regulated alternative splicing via CLIP-seq in human embryonic stem cells and found a large cohort of targets [51]. Supporting further FOX2 function as a critical regulator of splicing networks, many cancer- and FOX-regulated splicing events reported by Venables and collaborators affected genes associated with actin filaments, myosin dynamics, kinesins, and microtubule binding and trafficking complexes [33]. Therefore, it appears that splicing alteration in breast cancer may have a marked effect on genes involved in cellular architecture, plasticity, and movement.

 This observation is likely relevant to other types of cancers as a large number of differentially spliced genes when comparing lung, digestive tract, and brain tumors to normal tissues were associated with cell motility and organization of the actin cytoskeleton (Table 1). These observations suggest that genes involved in cellular architecture, plasticity, and movement are particularly prone to alternative splicing and/or that tumor progression results in or requires changes in the splicing patterns of genes involved in these cellular programs. It must be underlined that genes involved in other cellular programs, such as cell proliferation, are also often found to be differentially spliced in tumors (Table 1). Although further genome-wide analyses of alternative splicing are still required, an emerging concept is that cellular programs altered during tumor initiation and progression depend on splicing programs (Figure 3).

## 3. Cellular Programs Involve Splicing Programs Controlled by Splicing Factors

The first demonstration that splicing programs participate in cellular programs came from the study of the neuronal splicing factor, Nova-1, that was shown to regulate splicing events of a network of genes involved in synapse function [52, 53]. In the context of cancer-related cellular programs, the best example illustrating this concept was provided by a recent study of Warzecha and collaborators, who uncovered a network of alternative splicing changes that are under the control of the ESRP1 (RBM35A) and ESRP2 (RBM35B) splicing factors and that contribute to the epithelial-mesenchymal transition (EMT) [54]. Epithelial splicing regulatory proteins 1 and 2 (ESRP1 and ESRP2) have been identified to be regulators of the epithelial to mesenchymal splicing pattern of FGFR2, CD44, CTNND1 (p120-Catenin), and ENAH transcripts [55]. Warzecha and collaborators demonstrated that ESRPs are components of an epithelial gene signature and demonstrated a downregulation of these proteins in cells that undergo EMT. Knockdown of ESRPs results in loss of characteristic morphological features of epithelial cells with an increased motility and expression of invasive markers, concomitant with changes in expression of several prototypical EMT markers [54]. To know if ESRPs are master regulators of epithelial cell-specific splicing program, they analyzed splicing profiles derived from ectopic expression of ESRP1 in mesenchymal cells, as well as from knockdown of ESRPs in epithelial cells using splicing sensitive microarrays [54, 56]. They identified hundreds of alternative splicing events within numerous genes with functions in cell-cell adhesion, polarity, and migration, and many events showed reciprocal changes in the two experimental conditions. Components of this global ESRP-regulated epithelial splicing program could be valuable molecular markers to characterize the EMT and could have potential clinical applications, as cancer cells undergoing EMT present more aggressive tumor phenotypes.

Other cellular programs that may involve splicing programs are cell proliferation and apoptosis, two key cellular programs that are altered in cancer [14, 57]. Strikingly, most (if not all) genes involved in apoptosis, can produce splicing variants coding for protein isoforms with either pro- or antiapoptotic activities [16, 58]. Interestingly, an alternative splicing network that links cell cycle and apoptosis has recently been identified [59]. It must be emphasized that several cell cycle regulators, including CDC40 and CDC5L, have been identified as spliceosome components, and that several splicing factors, including SRSF1 (ASF/SF2, see below) affect cell cycle progression [60–62]. In addition, both splicing factors SRSF1 and SRSF2 (SC35) regulate the alternative splicing of several genes involved in apoptosis [59, 63–65]. The SRSF1 factor, a member of the arginine/serine-rich (SR) family of splicing factors, is particularly interesting as it is overexpressed in various human tumors [66, 67], and its overexpression is sufficient to transform immortalized cell lines [66]. Karni and collaborators showed that SRSF1 affects the alternative splicing of the tumor suppressor BIN1, producing an isoform lacking tumor suppressor activity, and of the protein kinases MNK2 and S6K1, leading to an MNK2 isoform promoting MAPK-independent eIF4E phosphorylation and an S6K1 isoform with oncogenic properties [66]. The oncogenic activity of SRSF1 may also be due to its implication in multiple cellular programs, as it regulates the alternative splicing of genes implicated in proliferation (e.g., CyclinD1), apoptosis (e.g., Bcl-x and Mcl1) and cell motility (e.g., Rac1 and Ron) [59, 68]. These studies provide evidence that an abnormally expressed splicing factor can have oncogenic properties by impacting on alternative splicing of cancer-associated genes and genes involved in cancer-related cellular programs. 

Also interesting in this context is the Sam68 (Src associated in mitosis, of 68 kDa) factor that is a KH domain RNA-binding protein, whose expression and function have been linked to the onset and progression of tumors, such as prostate and breast carcinomas [69–73]. It was first thought that Sam68 had a tumor suppressor role [74, 75], but direct investigations rather suggest a pro-oncogenic role of the protein (for review [76]). Sam68 regulates splicing events in several genes involved in apoptosis and cell proliferation (e.g., Bcl-x, Cyclin D1, and CD44) [77–80]. Interestingly, Valacca and collaborators suggested that Sam68 could also contribute to the malignant transformation of epithelial cancers by regulating the alternative splicing of SRSF1 and inducing EMT [79].

In addition to the splicing factors mentioned above including FOX2, the polypyrimidine tract-binding protein (PTB/PTBP1), also known as hnRNP I is a splicing regulator that often acts as a repressor, although it can also promote the inclusion of exons [81, 82]. PTB has been shown to be overexpressed in ovarian cancer and in gliomas and may play a role in tumor initiation and progression [83–86]. PTB is expressed throughout development, and then down-regulated in many adult tissues [87]. In ovarian and glioma cell lines, loss of PTB inhibited cell proliferation and cell migration and increased cell adhesion [83, 85]. Cheung and collaborators showed that after removal of PTB in glioma cell lines where the PTB paralog nPTB/PTBP2 is also expressed, a single gene RTN4 had enhanced inclusion of exon 3 and this isoform decreased cell proliferation, migration, and adherence to a similar degree as the removal of PTB [83]. Importantly, it has also been shown recently that PTB, together with two others hnRNP family members, hnRNPA1 and hnRNPA2, controls the alternative splicing of transcripts of the PKM gene coding for the enzyme pyruvate kinase. The expression of these proteins favors switching from PKM1 to PKM2 isoform, which promotes aerobic glycolysis and thus will provide a selective advantage for tumor formation [84, 88]. Further studies will be required to assess the potential relevance of other PTB-regulated exons in the context of cancer.

## 4. Conclusions

Genome-wide analyses of alternative splicing allow us to propose a model whereby splicing programs, together with transcription programs participate in the corruption of cellular programs during tumor initiation and progression (Figure 3). It is important to underline that recent proteomic analyses confirm the presence of alternative protein isoforms in tumor samples [89]. Because of the diversity generated by alternative splicing and because of its highly dynamic regulation, it is likely that this process plays a central role in the phenotypic plasticity of tumor cells. Therefore, the identification of splicing programs that impact on key cellular programs will allow a better understanding of the genetic programs involved in cancer initiation and progression. This concept likely applies to other cancer-related cellular programs in addition to EMT, migration, proliferation, and death. For example, angiogenesis (blood vessel formation) favors tumor progression by improving tumor cell feed [14]. Several alternative splicing events induce a switch from pro- to antiangiogenic functions [90, 91]. Likewise, there is increasing evidence that primary metabolism is altered in tumor cells, and the pyruvate kinase M1 and M2 splicing isoforms control the balance between aerobic and anaerobic glycolysis during tumor progression [92, 93]. Supporting a model where cellular metabolism could depend on splicing programs, it has been recently shown that several key regulators of cholesterol biosynthesis and uptake are regulated by alternative splicing in a coordinated manner by the splicing factor PTB/hnRNP I [94].

A current challenge raised by large-scale analyses of alternative splicing is the functional analysis of large sets of splice variants that are identified as misregulated in cancer. Most studies so far have relied on the functional annotation of genes and on the analysis of functional features encoded by alternative exons (e.g., protein domains or premature stop codons). However, there is a need for midscale functional analyses of splice variants using experimental screens. Such an approach was used in a recent study, where 41 splice variants previously associated with breast and/or ovarian cancer were functionally analyzed by transfecting cells with either isoform-specific siRNAs or bifunctional-targeted oligonucleotides allowing to reprogram alternative splicing, followed by analysis of cell growth, viability, and apoptosis assays [95]. In this pioneering study, about 10% of the analyzed splice variants were found to play a role in cell viability in the growth conditions that were used. While it is possible that only a subset of cancer-associated splice variants play a role in cancer cell phenotypes, it is likely that unraveling the functions of cancer-associated splice variants will require using more varied growth conditions and phenotypic screens involving various cellular programs.

Understanding the mechanisms leading to splicing program alteration in cancer will require identification of the splicing factors that govern these splicing programs, knowing that many of them are misregulated in cancer. While several splicing factors have recently emerged as good candidates, further studies are needed to identify the whole sets of splicing events they regulate, their precise cellular functions or their potential alterations in cancer. Moreover, it is likely that many other splicing factors will be involved in oncogenesis and tumor progression.

Finally, the identification of the splicing factors controlling cancer-associated splicing programs will allow developments of new therapeutic targets [96, 97]. In this context, several strategies targeting splicing regulators are currently being developed. The recent identification of small molecules that can inhibit the activity of splicing factors will allow improved targeted cancer therapies [98, 99]. However, as splicing factors are RNA-binding proteins that are often involved in other aspects of RNA metabolism (e.g., mRNA stability and translation, or processsing of noncoding RNAs), it will be important to determine whether these different activities cooperate in the induction of a given cellular program or can be selectively targeted to induce distinct phenotypes.

## Figures and Tables

**Table 1:** Summary of studies using different approaches (1: high throughput RT-PCR, 2: Affymetrix junctions arrays; 3: Affymetrix exon arrays; 4: exonhit arrays; 5: custom arrays) to identify misregulated gene at the splicing level in cancer. Bioinformatic gene pathway analyses.

	Samples	#	Genes functions	References
	Pooled human normal and tumor samples	1	*Cellular architecture, plasticity and movement	[33]
	26 human breast cancer cell lines and 5 nonmalignant immortalized cell lines	2	*Signaling (axon guidance, ephrin receptor, integrin, and tight junctions), cytoskeleton organization, biogenesis, and cell signaling	[34]
Breast	Nonmetastatic (67NR, 168FARN) and metastatic (4T07, 4T1) mouse primary tumors	3	*Cellular morphology, and cellular movement	[35]
	168FARN, 4T07 and 4T1 mouse primary tumors	3	*Cell growth, cell interactions, cell proliferation, cell migration, cell-to-cell signaling, cell death	[36]
	120 human breast tumors and 45 benign lesions	4		[37]

	18 paired samples of human lung tumors and normal adjacent tissues	3	Remodeling of the cytoskeleton and cell movement	[38]
Lung	20 paired of human primary lung tumors and adjacent normal tissues	3	*Tissue development, cellular growth and proliferation, tissue morphology, and immune response	[39]
	20 paired of human primary lung tumors and adjacent normal tissues	5	Cell adhesion, differentiation, proliferation, adhesion, migration, cytoskeleton, trafficking	[40]
	29 paired of human primary lung tumors and adjacent normal tissues	5	Cell signaling, cell proliferation, angiogenesis, cytoskeleton	[41]

	10 paired of human colon primary tumors and adjacent normal tissues	3	*Cell motility and organization of the actin cytoskeleton, cell adhesion, and matrix organization	[42]
Digestive tract	20 human colon adenocarcinoma and 10 normal samples	3	Cancer-related, cytoskeleton, matrix organization, Wnt signaling	[43]
	12 samples of isolated cells from 10 patients	3		[44]
	83 human colorectal tissue samples	3		[45]

	14 pediatric medulloblastomas and 5 samples of normal cerebellum	3	Neuronal differentiation, cancer progression: cytoskeleton remodeling, cell morphology regulation, and cell-to-cell interaction	[46]
Brain	47 human neuroblastoma samples in stage 1 and stage 4 with normal or amplified MYCN copy number	3	*Nervous system development, cell adhesion, synaptic transmission, and cytoskeleton organization and biogenesis	[47]
	24 human glioblastoma and 12 nontumor samples	3	Splicing, intracellular transport and cell migration, central nervous system, notch signaling, cell adhesion, apoptosis, cell growth	[48]
	26 human glioblastoma, 22 oligodendrogliomas and 6 nontumor samples	3		[49]
